# The C291R Tau Variant Forms Different Types of Protofibrils

**DOI:** 10.3389/fnmol.2020.00039

**Published:** 2020-03-18

**Authors:** Thomas K. Karikari, Rachel Thomas, Kevin G. Moffat

**Affiliations:** ^1^School of Life Sciences, University of Warwick, Coventry, United Kingdom; ^2^Midlands Integrative Biosciences Training Partnership, University of Warwick, Coventry, United Kingdom; ^3^Department of Psychiatry and Neurochemistry, Institute of Neuroscience and Physiology, The Sahlgrenska Academy, University of Gothenburg, Gothenburg, Sweden

**Keywords:** *MAPT* mutations, tau C291R, corticobasal degeneration, granular oligomer, annular protofibril, linear protofibril, atomic force microscopy, transmission electron microscopy

## Abstract

Mutations in the *MAPT* gene can lead to disease-associated variants of tau. However, the pathological mechanisms behind these genetic tauopathies are poorly understood. Here, we characterized the aggregation stages and conformational changes of tau C291R, a recently described *MAPT* mutation with potential pathogenic functions. The C291R variant of the tau four-repeat domain (tau-K18; a functional fragment with increased aggregation propensity compared with the full-length protein), aggregated into a mix of granular oligomers, amorphous and annular pore-like aggregates, in native-state and heparin-treated reactions as observed using atomic force microscopy (AFM) and negative-stained electron microscopy. On extended incubation in the native-state, tau-K18 C291R oligomers, unlike wild type (WT) tau-K18, aggregated to form protofibrils of four different phenotypes: (1) spherical annular; (2) spherical annular encapsulating granular oligomers; (3) ring-like annular but non-spherical; and (4) linear protofibrils. The ring-like tau-K18 C291R aggregates shared key properties of annular protofibrils previously described for other amyloidogenic proteins, in addition to two unique features: irregular/non-spherical-shaped annular protofibrils, and spherical protofibrils encapsulating granular oligomers. Tau-K18 C291R monomers had a circular dichroism (CD) peak at ~210 nm compared with ~199 nm for tau-K18 WT. These data suggest mutation-enhanced β-sheet propensity. Together, we describe the characterization of tau-K18 C291R, the first genetic mutation substituting a cysteine residue. The aggregation mechanism of tau-K18 C291R appears to involve β-sheet-rich granular oligomers which rearrange to form unique protofibrillar structures.

## Introduction

Tau protein is a product of the microtubule-associated protein tau (*MAPT*) gene located on chromosome 17q21 (Neve et al., 1986; Andreadis et al., 1992). *MAPT* is comprised of 16 exons, producing six tau isoforms in the adult human brain (Goedert et al., 1988, 1989). Tau has two significant components: the N-terminus projection domain and the assembly domain covering the microtubule-binding region (MTBR) and the C-terminus region (Andreadis et al., 1992; Andreadis, 2005). Variations in the number of N-terminus domains (0, 1 or 2) and MTBR domains (3 or 4 repeats) are the defining features of different isoforms (Andreadis et al., 1992; Andreadis, 2005). Notably, alternative splicing of exon 10 affects the ratio of three- to four-repeat tau isoforms, changes in which have been linked to several tauopathies (Liu and Gong, 2008). Indeed, many *MAPT* mutations located in or around exon 10 have been reported from individuals affected by different genetic tauopathies, with over a dozen implicated in disease (Goedert and Jakes, 2005; Ghetti et al., 2015). However, until recently, none of the described mutations affected a cysteine residue. Each tau isoform has either one or two cysteine residues, depending on the number of repeat domains in the MTBR: four-repeat isoforms have two cysteine residues, at positions 291 and 322, whilst three-repeat isoforms have only cysteine-322. Therefore, cysteine-322 is ubiquitous to all tau isoforms whilst cysteine-291 is limited to four-repeat isoforms. Several studies have reported that the presence of the cysteine-291 residue is important for tau aggregation and that the cysteine-322 residue may be inhibitory to this process (Bhattacharya et al., 2001; Crowe et al., 2013; Soeda et al., 2015; Al-Hilaly et al., 2017).

**Figure 1:**
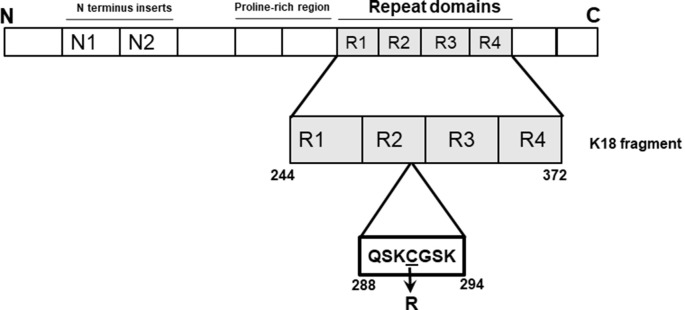
Schematic illustration of the tau-K18 C291R construct used in this study. The microtubule-binding region of tau (amino acids 244–372 of full-length tau-441; also known as tau-K18) was cloned into a pProEx-HTa plasmid and the C291R mutation introduced by site-directed mutagenesis and sequence-verified. This genetic construct was transformed into BL21(DE3)*pRosetta *Escherichia coli*, expressed and purified following a previously-described protocol also used for preparing wild type tau-K18 (Karikari et al., 2017).

In 2015, Marshall et al. (2015) identified a cysteine-modifying *MAPT* mutation altering cysteine-291 to arginine in a patient diagnosed with corticobasal degeneration (CBD) with apraxia of speech. This residue is sandwiched between two XSK tripeptide motifs (where X = Q or G; Figure 1), changing it to arginine (Marshall et al., 2015). The disease relevance of this potential genetic form of CBD as yet cannot be confirmed since it has neither been observed at post-mortem nor traced to any relative of the patient. However, given the importance of cysteine residues, particularly cysteine-291, to specific physiological and pathophysiological functions of tau, including acetyltransferase activity and aggregation (Schweers et al., 1995; Cohen et al., 2013; Soeda et al., 2015; Al-Hilaly et al., 2017; Chen et al., 2018), we were interested in understanding how the C291R mutation might affect tau aggregation. Importantly, the core of tau filaments isolated from CBD patient brains consist of a broad range of amino acids (amino acids 274–380 of full-length tau) covering cysteine-291 and the lysine residues that immediately surround it (Zhang et al., 2020). These lysine residues immediately flanking cysteine-291 on either side (lysine-290 and lysine-294) are thought to strengthen cysteine-291’s disulfide bonding capacity, a property critical to tau protein’s aggregation both *in vitro* and *in vivo* (Cisek et al., 2014). Indeed, the side chains of lysine-290 and lysine-294 are key components of an extra density structure within CBD filament folds (Zhang et al., 2020). We, therefore, hypothesized that substituting the hydrophobic cysteine-291 residue with arginine will generate a new stretch of basic amino acids that might lead to functional consequences on aggregation and conformation. In this study, we present the first biochemical characterization of tau C291R focusing on its step-wise aggregation stages, conformational and structural changes.

## Materials and Methods

### Cloning, Protein Expression and Purification

We generated the pProEx-HTa-Myc-6 × His-K18 plasmid carrying the C291R mutation (TGT > CGT, the same codon change previously reported by Marshall et al., 2015) by site-directed mutagenesis using a WT tau-K18 plasmid as a template (Karikari et al., 2017, 2019a). Protein expression and purification were achieved using our previously-characterized recombinant tau production protocols (Karikari et al., 2017, 2019a,b; Hill et al., 2019). The purified His-tagged tau-K18 constructs were used directly in functional experiments since the His-tag does not appear to affect aggregation (Huseby et al., 2019). Protein concentration was estimated using a Bicinchoninic acid assay kit from G-Biosciences (#786–570).

### Circular Dichroism (CD) Spectroscopy

A Jasco J-815 CD spectropolarimeter was used to collect CD spectra on 22 μg/ml of purified tau-K18 WT and tau-K18 C291R, each diluted in 10 mM sodium phosphate (NaPhos) buffer pH 7.4. The following conditions were used: cell path length = 1 mm, data pitch = 0.1 nm, response time = 1 s, wavelength range = 190 nm – 240 nm, scan speed = 100 nm/min, and high-tension voltage = ≤550 V. Averages of 10 spectral accumulations were analyzed.

### Heparin-Induced Aggregation Analysis

A 2:1 mass concentration ratio reaction mix of WT or C291R tau-K18 to heparin was prepared using 0.8 mg/ml of tau-K18 and 0.4 mg/ml of heparin in a total volume of 20 μl. The mixture was incubated at 37°C without shaking for 48 h to form fibrils. Five microliters of each sample were spotted on Formvar carbon-coated 300-mesh copper grids (#AGS162-6, Agar Scientific, Stansted, UK) and allowed 2 min to bind. The unbound sample was removed by blotting with filter paper and 5 μl of uranyl acetate added for 2 min. A JEOL JEM-2010 transmission electron microscope (TEM) was used to image the samples.

### Native-State Aggregation Time Course

The tau-K18 preparations were incubated in 10 mM NaPhos buffer pH 7.4 at 37°C for 0, 24, 72, 168, 216 and 314 h. At each time point 10 μl aliquots were removed, snap-frozen and stored at −80°C until further analysis by atomic force microscopy (AFM) as described below.

### AFM Analysis of Aggregation Stages

Tau-K18 samples (0.5 mg/ml) from the aggregation time course experiment were spotted on to freshly-cleaved 11 mm × 11 mm mica sheets (#AGG250-3, Agar Scientific, Stansted, UK). Once dried, the unbound protein was washed away with sterile water and the mica sheet air-dried and analyzed in AC Air Topography (tapping) mode on an Asylum Research MFP3D-SA instrument. A scan size of 20 μm, a scan rate of 1 Hz, set point of 590.49 mV, an integral gain of 3, x- and y- offsets of 0, points and gains of 256 and drive amplitude of 129.06 mV were used. The Igor Pro 6.37 image processing and programming software was used for image processing.

## Results

### Secondary Structure Profile of Tau-K18 C291R

**Figure 2:**
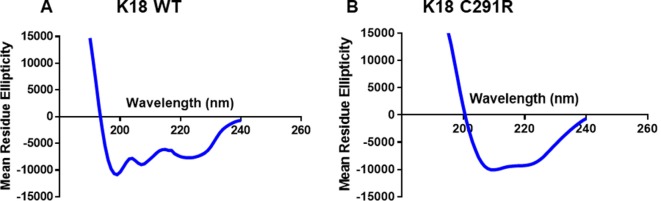
Circular dichroism (CD) profiles of tau-K18 WT **(A)** and C291R **(B)** monomers. Each construct was diluted in 10 mM NaPhos buffer pH 7.4 at 22 μg/ml final protein concentration and the secondary structure profiles analyzed as described in the “Materials and Methods” section.

Tau-K18 is generally unfolded but adopts β-sheet conformation with increasing aggregation (Kumar et al., 2014; Karikari et al., 2017). Specific *MAPT* mutations have distinct effects on this process by either increasing or decreasing the propensity to form β-sheet structures (Barghorn et al., 2000; Combs and Gamblin, 2012; Karikari et al., 2019a). To understand the possible effects of the C291R mutation on β-sheet formation, the secondary structure content of monomeric tau-K18 C291R and the WT were studied with CD spectroscopy. The experimental data in millidegrees were reconstructed in the Dichroweb database and the expected data (reconstructed minus experimental data) plotted in Figure 2. WT tau-K18 had a minimum peak of ~199 nm whilst tau-K18 C291R had a peak at ~210 nm (Figures 2A,B respectively), suggesting that the C291R variant has a higher propensity to adopt β-sheet conformation.

### Tau-K18 C291R Aggregates Into Amorphous and Spherical Structures, but Not Fibrils, Over 48 h in the Presence of Heparin

**Figure 3:**
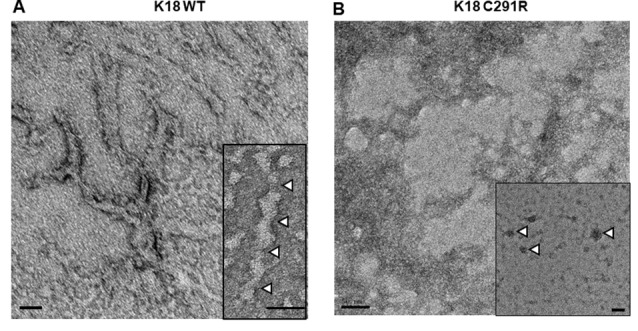
Negative-stain transmission electron microscope (TEM) micrographs of aggregates of tau-K18 WT **(A)** and C291R **(B)** formed after treating 0.8 mg/ml of each construct with 0.4 mg/ml of heparin and incubating for 48 h at 37°C without shaking. Panel **(A)** shows fibrils with the inset highlighting the periodic twists of ~50 nm (white arrowheads). Panel **(B)** shows amorphous aggregates, with the inset showing annular aggregates with defined outer structures (arrowheads). Scale bars = 50 nm for both main figures and insets.

As cysteine-291 is important for tau aggregation (Bhattacharya et al., 2001; Crowe et al., 2013; Soeda et al., 2015), we asked if the C291R mutation affects aggregation propensity. Each tau-K18 construct was mixed with a 50% mass concentration of heparin and incubated at 37°C without shaking for 48 h. Negative-stain TEM analysis showed that tau-K18 WT aggregated into fibrils (Figure 3A) in agreement with previous reports (Barghorn et al., 2000; Barghorn and Mandelkow, 2002; Karikari et al., 2017). These fibrils were 30–45 nm wide and 150–300 nm long. On the contrary, tau-K18 C291R mostly formed amorphous structures without any regular shape or form (Figure 3B) in addition to annular aggregates 20–50 nm wide (Figure 3B; inset). These results suggest that the C291R mutation averts tau-K18 aggregation into fibrils, but rather favors the formation of non-fibrillar oligomers and amorphous assemblies.

### Tau-K18 C291R Aggregates Into Non-fibrillar Amorphous and Annular Structures in Native-State Conditions

**Figure 4:**
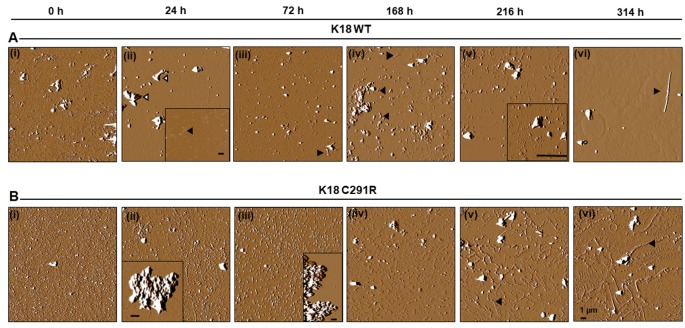
Atomic force microscopy (AFM) images of tau-K18 WT **(A)** and C291R **(B)** aggregates formed over 314 h in native state conditions (incubation at 37°C without shaking). WT tau-K18 aggregated into fibrils at 314 h (arrowhead) *via* granular oligomers and protofibrils intermediates recorded at earlier time points. The C291R variant initially aggregated into granular oligomers, amorphous aggregates and later into different types of protofibrils. Scale bars = 1 μm for all images. Further details about the intermediate structures may be found in the “Results” section.

To exclude the possibility that tau-K18 C291R’s aggregation into non-fibrillar structures was dependent on heparin induction, the experiment was repeated in native-state conditions by incubating both tau constructs in identical conditions as in Figure 3 except that no heparin was added. Aggregation was slower in this condition as anticipated (Karikari et al., 2019a). However, this approach allowed the fine mechanistic details of the process to be studied. AFM imaging revealed that tau-K18 WT monomers (Figure 4Ai) aggregated into granular oligomers (Figure 4Aii) and protofibril-like structures after 24 h (Figure 4Aii, inset). Clusters of aggregates identified at this stage (Figure 4Aii, white-filled arrowheads) were likely to be off-pathway structures that did not follow amyloidogenic aggregation. More filamentous structures (protofibrils and short fibrils) were observed at 72 h through 216 h (Figures 4Aiii–v, black arrowheads). *Bonafide* fibrils of up to 7 μm in length with paired helical filament (PHF) structure were recorded at 314 h (Figure 4Avi). This data shows that under careful conditions, tau-K18 WT can self-assemble into Alzheimer-like PHFs without the need for any external activation. Similar heparin-free aggregation studies have been performed for the recombinant form of a tau peptide (amino acid 297–391) that is a major component of PHF tau isolated from Alzheimer’s disease brains (Al-Hilaly et al., 2017, 2018, 2019).

**Figure 5:**
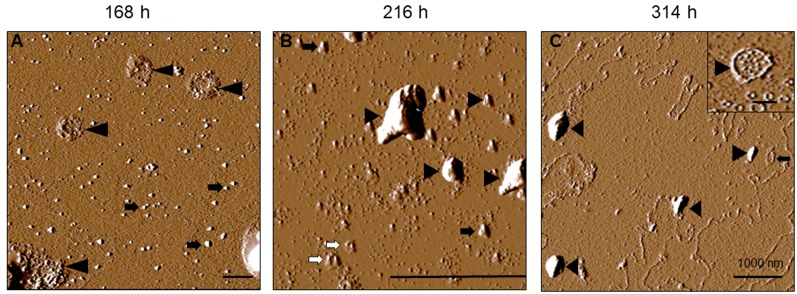
Increased resolution AFM images of tau-K18 C291R at later stages of the native-state aggregation process. Aggregate structures formed at 168 h **(A)**, 216 h **(B)** and 314 h **(C)** show granular oligomers (black arrows), amorphous aggregates (black arrowheads) and annular protofibrils including those encapsulating granular oligomers (inset in **C**). Scale bars = 1 μm.

For tau-K18 C291R, small-sized structures at 0 h (Figure 4Bi) shifted towards more granular orientation with increased sizes (5–10 nm) at 24 h, with large amorphous structures over 3 μm also evident at this stage (Figure 4Bii, inset). This coexistence of oligomers and amorphous aggregates was also observed at 72 h rather with increased oligomer sizes (Figure 4Biii). At 168 h, several medium-sized amorphous structures ~1 μm became evident (Figure 4Biv, arrowheads). Closer inspection showed that these amorphous structures contained granular oligomers (Figure 5A, arrowheads), suggesting that some oligomers were either absorbed into the amorphous structures or the oligomers rearranged to form these large structures. Regular granular oligomers were also visible (Figure 5A, arrows), signifying the coexistence of two distinct types of aggregates i.e., granular oligomers and amorphous structures. At 216 h, granular oligomers became less prominent but the medium-sized amorphous structures prevailed (Figure 4Bv). In addition, new structures with well-defined traces were recorded, which at higher resolution were made of various-sized amorphous clumps and groups of granular oligomers apparently in the process of forming amorphous clumps (Figure 5B, black arrowheads and arrows, respectively). Moreover, small ring-like annular structures made of granular oligomers were recorded beginning to take shape at 216 h (Figure 5B, white arrows). By 314 h, larger ring-like structures including those enclosing granular oligomers were observed (Figures 4Bvi, 5C inset). The structures with well-defined traces formed at late time points were similar to the amorphous aggregates imaged in negative-stained conditions in Figure 3B. Together, these results indicate that the C291R mutation leads to drastic changes in the aggregation pathway and products of tau-K18, suggesting an altered conformational effect of the mutation.

### Tau-K18 C291R Forms Different Types of Protofibrils

**Figure 6:**
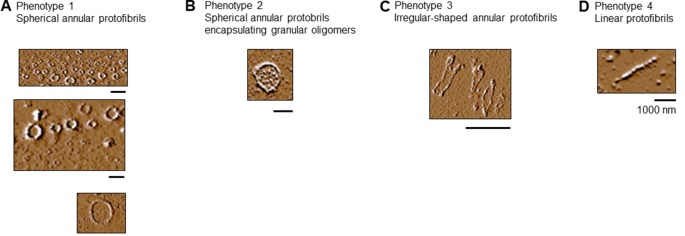
Tau-K18 C291R forms linear and annular protofibrils of distinct phenotypes. **(A)** Classical spherical annular protofibrils, **(B)** spherical annular protofibrils encapsulating dispersed granular oligomers, **(C)** annular aggregates of undefined shape, and **(D)** classical linear protofibrils. Scale bars = 1 μm.

We characterized the C291R annular protofibrils formed at 314 h binning into four phenotypes, namely:

1. *Spherical protofibrils*: protofibrils consisting of granular oligomers arranged in a spherical manner. The sizes (diameter) of these aggregates ranged from 50 to 100 nm, as well as up to over 1,000 nm (Figure 6A).
2. *Spherical annular protofibrils encapsulating granular oligomers*: in addition to being made of granular oligomers arranged in a spherical, ring-like structure, these aggregates consisted of single granular oligomer units dispersed within the enclosed structure (Figure 6B). These annular protofibrils were less frequently observed compared to the spherical ones above.
3. *Ring-like annular but non-spherical protofibrils*: These aggregates consisted of granular oligomers arranged in a non-spherical (often undefined) orientation (Figure 6C). The main similarity between this and the spherical protofibrils (phenotypes 1 and 2) is that they always formed a close-loop.
4. *Linear protofibrils*: these consisted of linearly arranged granular oligomers (Figure 6D), and differed from the other types of protofibrils by their arrangement compared to the ring-like structures of the others.

The frequency distribution of the protofibrils at 314 h, based on structure counts from AFM images from three experiments, can be found in Table 1.

**Table 1:** Frequency distribution of protofibrils phenotypes for tau-K18 C291R.

Phenotype	Frequency (n, %)
Spherical	223/329 (67.8%)
Linear	56/329 (17.0%)
Ring-like annular but non-spherical	42/329 (12.8%)
Spherical encapsulating oligomers	8/329 (2.4%)

## Discussion

*In vitro* biochemical studies provide critical molecular insights into potential disease-causing mechanisms of *MAPT* mutations identified in humans affected by specific tauopathies including effects on tau aggregation, phosphorylation, and microtubule-binding (Ghetti et al., 2015; Strang et al., 2019). However, little is known about *MAPT* mutations discovered more recently. In this study, we have provided the first biochemical characterization of the C291R tau variant, reported to have potentially pathogenic functions (Marshall et al., 2015). Cysteine-291 is a core component of CBD tau filament structure (Zhang et al., 2020), hence mutagenic changes are likely to have potential effects on aggregation properties. Our results demonstrate that the C291R mutation leads to a striking distinction in the aggregation mechanism of tau-K18 compared with the WT. Whilst WT tau-K18 underwent classical aggregation by progressively forming granular oligomers, protofibrils and fibrils, the C291R variant formed a mix of aggregates of three different major types: (1) granular oligomers, (2) amorphous aggregates of various sizes and shapes, and (3) protofibrils—both linear and annular structures made of granular oligomers. It is noteworthy that these observations were made in both heparin-induced and native state aggregation reactions and imaged using two different methods—negative-stained TEM and label-free AFM. Importantly, we did not record obvious fibrillar aggregates for tau-K18 C291R but observed that in addition to regular linear protofibrils, this variant formed annular aggregates with unique features. Together, these lines of evidence are consistent with our hypothesis that the C291R mutation results in an altered aggregation pathway with aggregate conformers of distinct structural properties compared with WT tau-K18 aggregates.

The aggregation of tau-K18 C291R into a mix of granular oligomers, annular structures, and amorphous aggregates appears to be an *off-pathway process* with respect to fibril formation (Ding et al., 2002; Lasagna-Reeves et al., 2014). Key properties of this non-fibrillar pathway were clearly identified from our AFM analysis: the process begun with the formation of granular oligomers, which further polymerized into amorphous aggregates of various sizes and, at later time points, annular structures. The annular aggregates were made of granular oligomers arranged in a ring-like shape with defined outer structures. These properties easily differentiated them from the amorphous structures (see Figures 4B, 5C) and regular granular oligomers (Kayed et al., 2009). Due to their defined multi-oligomer presentation, annular aggregates are often referred to as annular protofibrils, pore-like structures or pore-forming aggregates, and have been widely characterized for amyloid-beta and alpha-synuclein (Ding et al., 2002; Lashuel et al., 2002; Kayed et al., 2009), and in a single report for tau (Lasagna-Reeves et al., 2014). In the case of tau, annular aggregates were identified, using TEM, AFM, and a conformation-specific antibody, in *post mortem* brains of dementia with Lewy body and progressive supranuclear palsy patients. Moreover, annular aggregates were reported from brain isolates of P301L tau-expressing mice, but not WT mice, suggesting an association with specific *MAPT* mutations (Lasagna-Reeves et al., 2014).

In the present study, we have demonstrated that a new, potentially pathogenic variant of tau, C291R, also forms annular aggregates *in vitro* in addition to linear protofibrils. Importantly, the annular aggregates shared the following striking similarities with those described previously for tau, amyloid-beta, and alpha-synuclein: (1) their formation was preceded by spherical/granular oligomers; (2) the annular aggregates consisted of granular oligomers often arranged in a spherical fashion; (3) spherical annular aggregates were in a range of sizes; (4) annular aggregates were associated with a specific pathogenic mutation; and (5) pore-like annular protofibrils were clearly distinguishable from linear protofibrils which usually proceed to form fibrils (Ding et al., 2002; Lashuel et al., 2002, 2003; Lasagna-Reeves et al., 2014). In addition, two unique phenotypes of the tau-K18 C291R annular aggregates previously not described for an amyloidogenic protein/peptide were characterized, namely: (1) the spherically arranged granular oligomers that formed the annular aggregates occasionally enclosed small, single-unit granular oligomers within their larger ring structures (Figure 5C inset and Figure 6B); and (2) the ring-like shape of annular aggregates did not always have a spherical appearance. In fact, the shape was sometimes irregular and undefined (Figures 4Bv,vi, 5C, 6C). Together, we have demonstrated that the ring-like tau-K18 C291R aggregates possess key properties of annular protofibrils previously described for other amyloidogenic proteins. The new phenotypes presented to warrant further characterization in future studies.

Previous reports showed that substituting cysteine-291 with alanine (C291A) in WT tau forms affects the aggregation propensity, showing this reduces oligomer formation (Kim et al., 2015; Soeda et al., 2015; Al-Hilaly et al., 2017), demonstrating also that this does not prevent fibril formation (Barghorn and Mandelkow, 2002; Furukawa et al., 2011). Similarly, modifying cysteine-291 to serine (C291S) reduced its oligomerization competence (Kim et al., 2015). Together, these studies suggested that substituting cysteine-291 with Ala or Ser (both aliphatic amino acids) have similar effects. Moreover, we and others have shown that the C291A/C322A/I260C triple-modified K18 variant aggregates into both oligomers and fibrils (Kumar et al., 2014; Michel et al., 2014; Shammas et al., 2015; Karikari et al., 2019a,b). However, none of these modifications led to the formation of annular aggregates. Curiously, the C291A and C291S mutations both decreased aggregation competence in a manner distinct from the current C291R with a polar amino acid introduced, suggesting the choice of amino acid makes an important difference. Notably, the introduction of arginine at cysteine-291 had adverse effects on fibrillization processes but not oligomer formation as the C291R construct readily formed oligomers. We propose that the C291R mutation selectively impairs the conformational changes leading from oligomers to fibrils but not from monomers to oligomers. Cysteine residues in tau are surrounded by lysine residues whose basic charges enable disulfide bonding which is critical for aggregation (Lutolf et al., 2001). Removing one of the two cysteine residues in tau-K18 weakens its inter-molecular disulfide binding and hence aggregation tendency. The veracity of these hypothetical models need to be tested experimentally.

Although tau-K18 C291R did not form *bona fide* fibrils in the present study, we cannot rule out that the same construct can form fibrils under different experimental conditions (e.g., using different buffers and aggregation-inducing agents). Similar to our findings that linear and annular protofibrils of tau C291R were jointly present in the same reaction (see Figure 6), annular and fibrillar alpha-synuclein aggregates have been shown to co-exist in the same conditions (Shtilerman et al., 2002). Furthermore, tau P301L can form fibrils as well as annular aggregates *in vitro* and *in vivo* (Lasagna-Reeves et al., 2014), suggesting a high potential for co-existence.

A limitation of this study is that the proposed pathogenicity of the C291R mutation has not been confirmed by neuropathological examination. Therefore, the mutation and its functional properties described in this manuscript cannot be presently linked to CBD or any other form of tauopathy. Nonetheless, the results provide insights into the functional and structural properties of this novel mutation and sheds new light on the biochemical basis of tau aggregation. Moreover, reported differences in aggregate structures of human brain-derived tau and recombinant tau aggregated *in vitro* by inducing with heparin (Zhang et al., 2019) may limit the utility of the findings presented in this study. However, a more recent study showed close similarities between brain-derived tau filaments and those formed by the amino acid 297–391 fragment self-assembled *in vitro* without any external inducing agents (Al-Hilaly et al., 2019). Given that the tau-K18 C291R construct studied herein could also self-aggregate and contains about half of the tau 297–391 sequence, the findings of this study are likely to have potential implications for future *in vivo* and neuropathological studies.

## Conclusion

We have presented the first biochemical characterization of tau C291R, covering its secondary structure, step-wise aggregation stages, and conformational properties. A novel, significant finding is that tau C291R forms annular, pore-like aggregates, the second time this phenomenon has been described for a tau construct after tau P301L. Importantly, the C291R annular aggregates share major properties described for such structures formed by amyloid-beta, alpha-synuclein and tau P301L but also has two unique features previously unknown: (1) spherically arranged granular oligomers that enclose small, single-unit granular oligomers; and (2) non-spherical ring-like annular aggregates. These findings, and their further characterization in future studies, should pave the way for further studies into the C291R tau variant and how the annular aggregates it forms could provide new insights into the aggregation process of tau.

## Data Availability Statement

All datasets generated for this study are included in the article.

## Author Contributions

TK and KM conceived and co-ordinated the study and wrote the manuscript. TK and RT performed the experiments and analyzed the data. All authors critically revised and agreed on the final manuscript.

## Conflict of Interest

The authors declare that the research was conducted in the absence of any commercial or financial relationships that could be construed as a potential conflict of interest.

## Abbreviations

AFM: atomic force microscopy
CBD: corticobasal degeneration
CD: circular dichroism
C291A: cysteine-291 substituted with alanine
C291R: cysteine-291 substituted for arginine
C291S: cysteine-291 substituted for serine
MTBR: microtubule-binding region
NaPhos: sodium phosphate
TBS-T: Tris-buffered saline plus Tween
TEM: transmission electron microscopy
WT: wild type.
